# AG36 Inhibits Human Breast Cancer Cells Proliferation by Promotion of Apoptosis *In vitro* and *In vivo*

**DOI:** 10.3389/fphar.2017.00015

**Published:** 2017-01-26

**Authors:** Li-Hua Mu, Yu-Ning Wang, Dong-Xiao Wang, Jing Zhang, Li Liu, Xian-Zhe Dong, Yuan Hu, Ping Liu

**Affiliations:** ^1^Department of Clinical Pharmacology, Chinese PLA General HospitalBeijing, China; ^2^Department of Clinical Surgery, Chinese PLA General HospitalBeijing, China; ^3^Department of Chinese Medicine, Shanxi University of Traditional Chinese MedicineTaiyuan, China

**Keywords:** *Ardisia gigantifoli*a stapf., breast cancer cells, antitumor, *in vivo*, *in vitro*

## Abstract

AG36 is the biotransformation product of triterpenoid saponin from *Ardisia gigantifolia* stapf. In this study, the antitumor activity and underlying molecular mechanisms of AG36 against human breast MCF-7, MDA-MB-231, and SK-BR-3 cancer cells were investigated. AG36 inhibited the viability of MCF-7, MDA-MB-231, and SK-BR-3 cells in a dose and time-dependent manner, with an IC_50_ of approximately 0.73, 18.1, and 23.4 μM at 48 h, respectively. AG36 obviously induced apoptosis and G2/M arrest of all the three breast cancer cells. Moreover, AG36 decreased the protein expression of cycle regulatory proteins cyclin B1 or cyclin D1. In MCF-7 and MDA-MB-231 cells, AG36 strongly increased the cleaved caspase-3 and -8 protein expressions, while in SK-BR-3 cells, AG36 only increased the protein expression of cleaved caspase-3. In all the three breast cancer cells, the ratio of Bax/Bcl-2 and cytosolic cytochrome *c* content increased significantly compared with control group. The death receptor-related proteins Fas/FasL, TNFR1, and DR5 were detected by Western blot, it showed that different breast cancer cells activated the death receptor-mediated extrinsic caspase-8 pathway through different receptors. In addition, the caspase-8 inhibitor z-IETD-fmk could significantly block AG36-triggered MCF-7 cells apoptosis. The *in vivo* studies showed that AG36 significantly inhibited the growth of MCF-7 xenograft tumors in BALB/c nude mice comparing with control. In conclusion, AG36 inhibited MCF-7, MDA-MB-231, and SK-BR-3 cells proliferation by the intrinsic mitochondrial and the extrinsic death receptor pathways and AG36 might be a potential breast cancer therapeutic agent.

## Introduction

**FIGURE 1:**
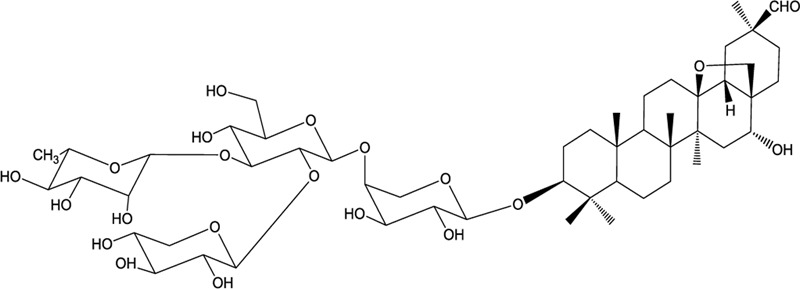
**Structure of AG36**.

The rhizome of *Ardisia gigantifolia* stapf. is a traditional Chinese medicine used as an expectorant for the treatment of traumatic injury, rheumatism, muscles, and bones pain. It is an evergreen dwarf shrub mostly distributed in the provinces of Guangxi, Jiangxi, and Fujian in China (Jiangsu New Medicinal College, 2001). According to previous studies, triterpenoid saponins from *A. gigantifolia* stapf. have shown antitumour activities (Li et al., 2009; Yokosuka et al., 2009; Gong et al., 2010; Mu et al., 2010, 2012). Among them, cyclamiretin A 3β-O-{α-L-rhamnopyranosyl-(1→3)-[β-D-xylopyranosyl-(1→2)]-β-D-glucopyranosyl-(1→4)-[β-D-gluco-pyranosyl-(1→2)]-α-L-arabinopyranoside} (AG4) had prom-inent cytotoxicity against MCF-7 cells (Zheng et al., 2013). In order to discover new anticancer lead compounds, AG4 was biotransformated by *Alternaria alternata* AS 3.6872 to obtain AG36 (Mu et al., 2015). The structure of AG36 is similar with that of AG4, but with four-sugar units at C-3 (**Figure 1**), and AG36 showed better cytotoxicity than AG4 against human breast cancer MCF-7 cells.

Breast cancer is one of the most common cancers for women worldwide (Guo et al., 2011). Globally, from 1980 to 2010, the incidence increased with an annual growth rate of 3.1%, breast cancer related mortality is still at a high level recently (Forouzanfar et al., 2011). The limitations associated with new therapeutic approaches for breast cancer, such as metastasis and relapse make breast cancer still a challenge (Baselga et al., 2012). In this study, we reported the anti-proliferative activity of AG36 against breast cancer cells *in vitro* and *in vivo* and unveiled the potential antitumor mechanisms of AG36. Our work provides experimental evidence for the medicinal applications of AG36 which may serve as a potential drug against human breast cancer.

## Materials and Methods

### Chemicals and Reagents

AG36 (purity: >99%) was the biotransformation product of triterpenoid saponin AG4 from *A. gigantifolia* stapf. as previously described (Mu et al., 2015). The Dulbecco’s phosphate buffered saline (DPBS), protease inhibitor cocktail, gelatin, and 3-(4,5-dimethylthiazol-2-yl)-2,5-diphenyltetrazolium bromide (MTT) were purchased from Sigma-Aldrich (St. Louis, MO, USA). The primary antibodies for cleaved-caspase-3, cleaved-caspase-8, deaved-caspase-9, Bax, Bcl-2, cytochrome *c*, TNFR1, Fas, FasL, DR5, and β-actin as well as all of the secondary antibodies were purchased from Cell Signaling Technology (Danvers, MA, USA).

### Cell Culture

The MCF-7 cell line was a kind gift of Prof. Ming Gang Bi from Institute of Medicinal Plant Development, Chinese Academy of Medical Sciences and Peking Union Medical College. The MDA-MB-231 and SK-BR-3 cell lines were purchased from Cell Culture Collection of Chinese Academy of Medical Sciences (Beijing, China). MCF-7 and SK-BR-3 cells were grown in Dulbecco’s modified Eagle’s medium (DMEM, Gibco) supplemented with 10% fetal bovine serum (FBS) and 1% penicillin/streptomycin in a humidified atmosphere containing 5% CO_2_ at 37°C. MDA-MB-231 cells were grown in L-15 medium containing 10% FBS at 37°C in non-CO_2_ conditions.

### Cell Viability Assay

The cell viability was evaluated by MTT assay. Briefly, MCF-7 cells were seeded at 2 × 10^4^ cells/well into 96-well plates and cultured in DMEM medium at 37°C for 24 h. The cells were then treated with final concentrations of AG36 (0, 0.2, 0.5, 1.0, and 1.5 μM) for 24, 48, and 72 h, respectively. MTT solution was added to each well and incubated for 4 h. The supernatant was aspired, and DMSO was used to dissolve the formazan crystals, and cellular viability was determined by measuring the absorbance at 570 nm by an enzyme-linked immunosorbent assay (ELISA) plate reader (Perkin-Elmer, Inc., 1420-012, China).

### Analysis of Cell Cycle by PI Staining

MCF-7 cells (1 × 10^5^/well) were seeded in six-well plates, treated by AG36 at various concentrations (0, 0.5, 1.0, and 1.5 μM) for 48 h, washed with PBS, and fixed with 70% (v/v) ethanol at 4°C for 1 h. The cells were washed with PBS twice and stained by 50 μg/mL PI and 10 μg/mL RNase A for 30 min in the dark (Fang et al., 2012). The cell cycle was measured using FACS Calibur flow cytometer (BD Biosciences, USA).

### Measurement of Cell Apoptosis

Apoptosis of cells was conducted using double staining with Annexin V-FITC and PI. After treatment by AG36 for 48 h, MCF-7 cells (AG36: 0, 0.5, 1.0, and 1.5 μM), MDA-MB-231 and SK-BR-3 cells (AG36: 0, 10, 15, and 20 μM) were collected and stained by Annexin V-FITC kit (Becton Dickinson, San Jose, CA, USA) Briefly, cells were washed twice with cold PBS and re-suspended in 300 μl binding buffer containing 10 μl Annexin V-FITC stock and 10 μL PI. The cells were incubated for 15 min at room temperature in dark and then analyzed using flow cytometry (FACS Calibur; Becton Dickinson, San Jose, CA, USA). In some experiments, caspase-8 inhibitor Z-IETD-FMK with final concentration of 10 μM was added into fresh medium of MCF-7 cells 1 h before AG36 was added. In some experiments, caspase-8 inhibitor Z-IETD-FMK with final concentration of 10 μM was added into fresh medium of MCF-7 cells 1 h before AG36 was added.

### Western Blot Analysis

For the Western blot analysis, after culture with AG36 for 48 h, MCF-7 cells (0, 0.5, 1.0, and 1.5 μM), MDA-MB-231 and SK-BR-3 cells (AG36: 0, 10, 15, and 20 μM) cells were collected by trypsinization and washed with cold PBS. The collected cells were lysed in total protein extraction reagent and proteinase inhibitors. The cell lysates were centrifuged at 12,000 × *g* for 15 min at 4°C. The protein concentration of the supernatants was determined by the BCA protein assay kit. Equal amounts of protein from each sample were separated on SDS-PAGE and transferred to a PVDF membrane. Membranes were blocked in 5% nonfat dry milk in TBST at room temperature for 1 h. Subsequently, the membranes were then washed three times and probed with different primary antibodies targeting cyclin B1, cyclin D1, cytochrome *c*, Bax, Bcl-2, caspase-3 caspase-8, caspase-9, FasL, Fas, DR5, and TNFR1 at 4°C overnight. The immunoblots were washed three times with TBST buffer and incubated with the HRP-conjugated secondary antibodies for 1 h at room temperature. The load protein was normalized to β-actin and the protein bands were enhanced with the enhanced chemiluminescence reagent (Pierce, Rockford, IL, USA).

### *In vivo* Xenograft Studies

Female BALB/c nude mice (5 weeks old, 18–19 g) were supplied by Beijing Vital River Laboratory Animal Co. Ltd. (Beijing, China). All care and procedures of all animal experiments were in accordance with the national guideline for the care and use of laboratory animals. Animals were inoculated with 2 × 10^6^ cells (0.1 ml/mouse) intraperitoneally (i.p.). Day “0” was assigned on tumor implantation day. On day 1, the animals were randomly divided into five different groups (*n* = 8). AG36 was administered i.p. at doses of 0.75, 1.5, and 3.0 mg/kg/day every 2 days. The CTX treated group was administered i.p. at dose of 25 mg/kg/day every 2 days. The control group was injected with the same volume of PBS instead. The tumor volumes were calculated using the following formula: tumor volume (mm^3^) = 0.56 × length (mm) × width^2^ (square mm). Body weights were recorded every 2 days to value the toxicity of AG36. Mice were sacrificed on the 17th day and the isolated tumors, livers, spleens, and kidneys were weighed.

### Statistical Analysis

All data were expressed as mean ± SD from three independent experiments. Data were analyzed statistically by ANOVA. Statistical comparisons were evaluated using Student’s *t*-test. Differences were considered to be significant at *P*-values less than 0.05.

## Results

### AG36 Inhibits Cell Viability and Proliferation in Breast Cancer Cells

**FIGURE 2:**
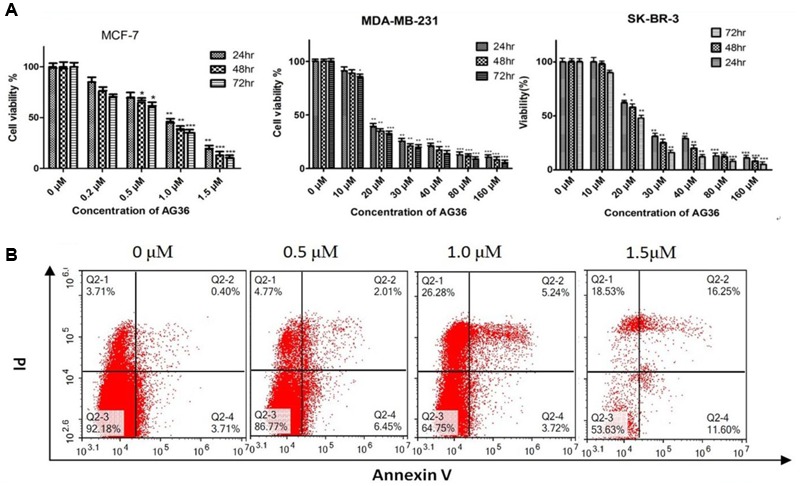
**(A)** Antiproliferation activity of AG36 on breast cancer cells at different time estimated with the MTT assay. Data were presented as mean ± SD of three independent experiments. ^∗^*P* < 0.05, ^∗∗^*P* < 0.01, and ^∗∗∗^*P* < 0.001 compared to the control group **(B)**. Flow cytometry detection of apoptosis with annexin V/PI in MCF-7 cells treated by AG36.

To screen the potential cytotoxic effect of AG36 against breast cancer, we examined the effect of AG36 on cell proliferation in MCF-7, MDA-MB-231, and SK-BR-3 cancer cells by MTT assay. As shown in **Figure 2A**, AG36 inhibited the viability of MCF-7, MDA-MB-231, and SK-BR-3 cells in a dose and time-dependent manner, with IC_50_ values of approximately 0.73, 18.1, and 23.4 μM at 48 h, respectively.

Based on the IC_50_ values, the AG36 concentrations of 0.5, 1.0, and 1.5 μM for MCF-7 and 10, 15, and 20 μM for MDA-MB-231 and SK-BR-3 were used in the following study. In order to determine whether decreased proliferation and cell viability were associated with apoptosis, the apoptotic effects of AG36 on MCF-7 cells were investigated using annexin-V/PI double staining by flow cytometry. As shown in **Figure 2B**, after 48 h treatment with AG36, the percentages of apoptotic cells of MCF-7 cells were from 7.8 to 46.3%. In MCF-7, AG36 noticeably reduced the surviving cells and increased the early and late apoptotic cells in a dose-dependent manner.

### AG36 Induces Cell Cycle Arrest

**FIGURE 3:**
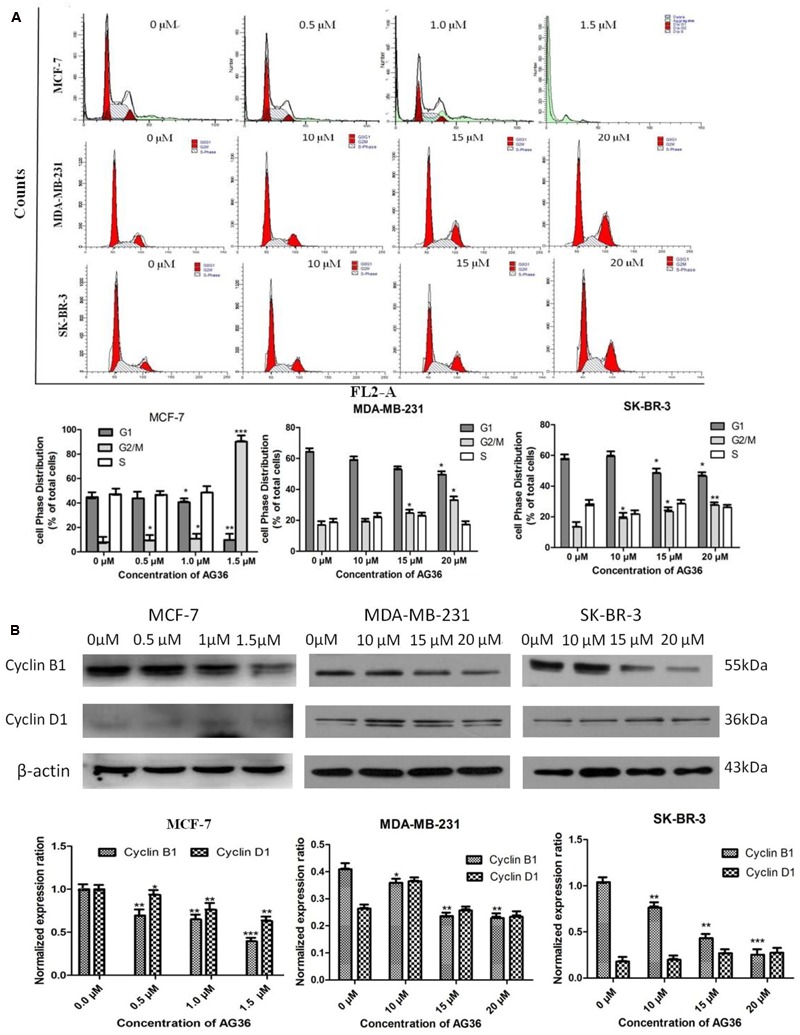
**AG36 induced cell cycle arrest in human breast cancer cells. (A)** Cells were treated with indicated concentrations of AG36 for 48 h and then were analyzed by flow cytometry. **(B)** Western blotting to examine the expression of cyclin B1 and cyclin D1 in MCF-7, MDA-MB-231, and SK-BR-3 cells treated with AG36 for 48 h. β-Actin expression was used as a loading control. All data are represented as means ± SD of three independent experiments ^∗^*P* < 0.05, ^∗∗^*P* < 0.01, and ^∗∗∗^*P* < 0.01.

Cell cycle arrest is a common mechanism for the cytotoxic effects of anticancer drug. To investigate the effect of AG36 on cell cycle arrest, DNA contents in different phases of MCF-7, MDA-MB-231, and SK-BR-3 cell cycle were performed by flow cytometry. Treatment with AG36 for 48 h, the number of cells was remarkably increased in G2/M phase with a concomitantly decrease in G1 phase compared to control (**Figure 3A**), indicating that AG36 could significantly inhibit DNA synthesis of MCF-7, MDA-MB-231, and SK-BR-3 cells. To understand the possible molecular events associated with AG36-induced cell cycle arrest in breast cancer cells, cell cycle regulatory proteins cyclin B1 and cyclin D1 were examined using Western blot analysis. Exposure to AG36 for 48 h strongly decreased the expression level of cyclin B1 and cyclin D1 in MCF-7 cells (**Figure 3B**). In MDA-MB-231 and SK-BR-3 cells, AG36 decreased the expression level of cyclin B1 significantly. These results indicate that AG36 can reduce breast cancer cell proliferation by G2/M-phase cell cycle arresting through the downregulation of cyclin B1 or cyclin D1.

### Effect of AG36 on Expressions of Cytochrome *c* and Bcl-2 Family Proteins

**FIGURE 4:**
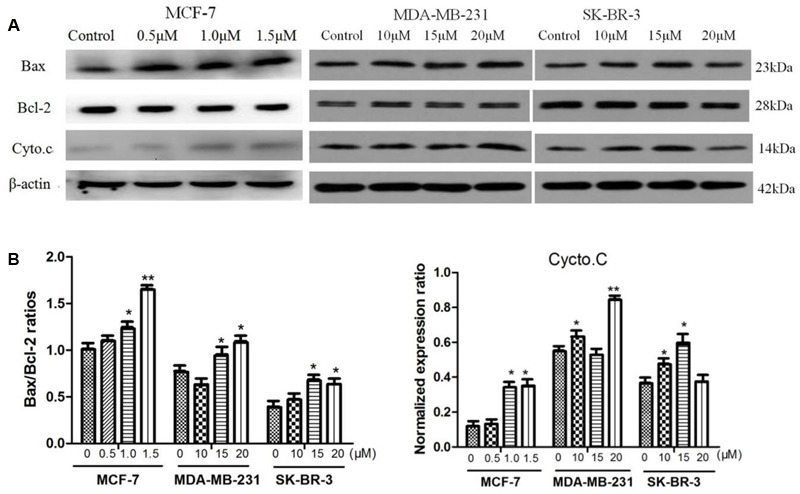
**Effect of AG36 on Bax, Bcl-2, and Cyto.c protein expression of breast cancer cells. (A)** Cells were treated with various concentrations of AG36 for 48 h. **(B)** The Bax/Bcl-2 ratio and cycto.C data were presented as mean ± SD of three independent experiments. ^∗^*P* < 0.05, ^∗∗^*P* < 0.01 compared to the control group.

The apoptotic-related proteins Bax and Bcl-2 play a crucial role in cell apoptosis (Arnoult et al., 2002). The cytochrome *c* release from the mitochondria is a necessary requirement to initiate apoptotic cell death pathway (Kakkar and Singh, 2007). The effects of AG36 on protein expressions of cytochrome *c*, Bax and Bcl-2 in MCF-7, MDA-MB-231, and SK-BR-3 cells were tested by Western blot analysis. AG36 can induce cytochrome *c* release from the mitochondria into the cytoplasm in the three breast cancer cell lines (**Figures 4A,B**). In MCF-7 and MDA-MB-231, AG36 increased the expression of Bax in a concentration-dependent manner, whereas Bcl-2 expression did not change apparently as compared with the control group. In SK-BR-3 cells, AG36 decreased the expression of Bcl-2 (**Figure 4A**). For all the three breast cancer cells, AG36 treatment increased the Bax/Bcl-2 ratio (**Figure 4B**), suggesting that Bcl-2 family proteins involved in AG36-induced apoptosis in breast cancer cells. These findings suggest that AG36 could induce apoptosis of MCF-7, MDA-MB-231, and SK-BR-3 cells by mitochondria-dependent pathway.

### Effects of AG36 on Caspase-3, -8, and -9 Activation

Caspases has been demonstrated to play a central role during cellular apoptosis (Sun et al., 1999; Wang et al., 2005). Caspase-3 is a prevalent caspase that is ultimately responsible for the majority of apoptotic processes and can be activated by upstream initiator caspases, such as caspase-8 or -9 through two distinct pathways, i.e., the death receptor-mediated extrinsic caspase-8 pathway or the mitochondria dependent-cytochrome *c*/caspase-9 intrinsic pathway, respectively (Budihardjo et al., 1999; Igney and Krammer, 2002; Hu and Kavanagh, 2003).

**FIGURE 5:**
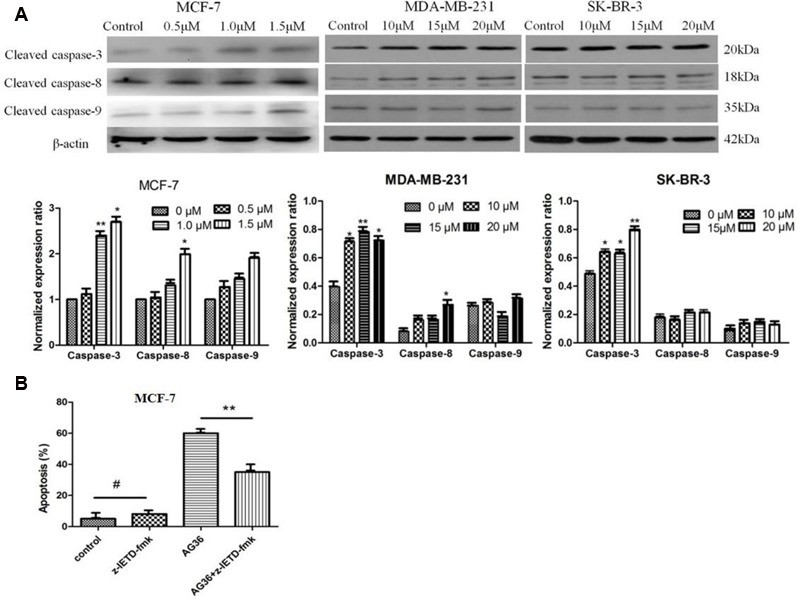
**(A)** Effects of AG36 on the expression and activation of caspase-3, 8, and 9 in different breast cancer cells. **(B)** Effects of caspase-8 inhibitors on AG36 induced cell death in MCF-7 cells. Data were presented as mean ± SD of three independent experiments. ^∗^*P* < 0.05, ^∗∗^*P* < 0.01 compared to the control group. #*P* < 0.01 compared to the AG36 group.

Therefore, we examined the effects of AG36 on the activation of caspase-3, -8, and -9. As shown in **Figure 5A**, AG36 increased the protein expression of cleaved caspase-3 and -8 in a dose-dependent manner in MCF-7 and MDA-MB-231 cells, whereas cleaved caspase-9 did not strongly increase in response to AG36 treatment, confirming the involvement of death receptor-mediated pathway in the AG36-induced apoptosis in MCF-7 and MDA-MB-231 cells. But in SK-BR-3 cells, AG36 dose-dependently increased the protein expression of cleaved caspase-3 without increasing the cleaved caspase-8 and -9 protein expressions. These results indicated that caspase-8 or -3 play pivotal roles in AG36-induced apoptosis of MCF-7, MDA-MB-231, and SK-BR-3 cells.

To further evaluate the role of caspases in the AG36-induced apoptosis pathway, we examined whether specific caspase-8 inhibitors, namely z-IETD-fmk block AG36-induced cellular apoptosis in MCF-7 cells. As shown in **Figure 5B**, z-IETD-fmk effectively inhibited AG36-induced MCF-7 cell apoptosis. These results indicated that AG36-induced apoptosis of MCF-7 cells was also dependent on the caspase-3 and -8 cascade activation.

### Effect of AG36 on Expressions of FasL, Fas, DR5, and TNFR1 Proteins

**FIGURE 6:**
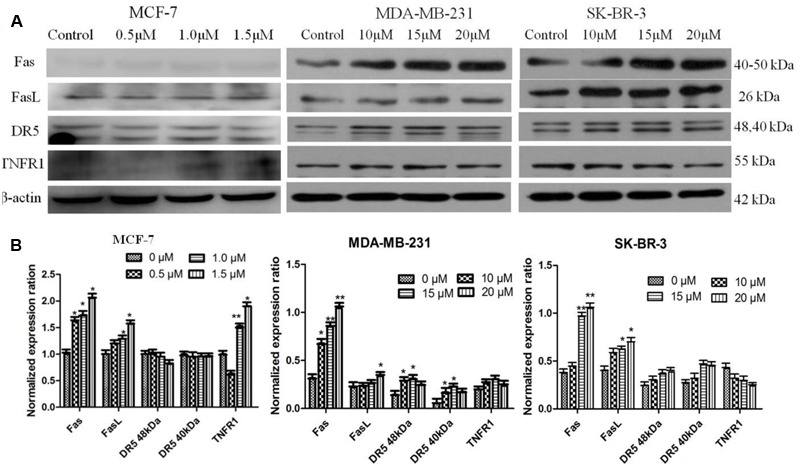
**Activation of FasL, Fas, DR5, and TNFR1 in AG36-treated breast cancer cells. (A)** Cells were treated with indicated concentrations of AG36 for 48 h. **(B)** Representative histograms for FasL, Fas, DR5, and TNFR1 expression in breast cancer cells. Data were presented as mean ± SD of three independent experiments. ^∗^*P* < 0.05, ^∗∗^*P* < 0.01 compared to the control group.

In order to test the effect of AG36 on death receptor signal pathway, we evaluated the contribution of FasL, Fas, DR5, and TNFR1 to apoptosis of MCF-7, MDA-MB-231, and SK-BR-3 cells by Western Blot (**Figure 6A**). When MCF-7 cells were treated with AG36 for 48 h, FasL, Fas, and TNFR1 were activated in a dose-dependent manner (**Figure 6B**), respectively, while DR5 levels did not change apparently, which suggested the activation of FasL/Fas and TNFR1-signaling apoptotic pathway as well as the downstream caspase cascade reaction. In MDA-MB 231 cells, as the dose of AG36 increased, levels of FasL/Fas and DR5 were upregulated, whereas levels of TNFR1 were almost unchanged. In SK-BR-3 cells, AG36 only increased the protein expressions of Fas and FasL without significantly affecting the levels of DR5 and TNFR1.

### Efficacy of AG36 to Inhibit Tumor Growth in Nude Mice

**FIGURE 7:**
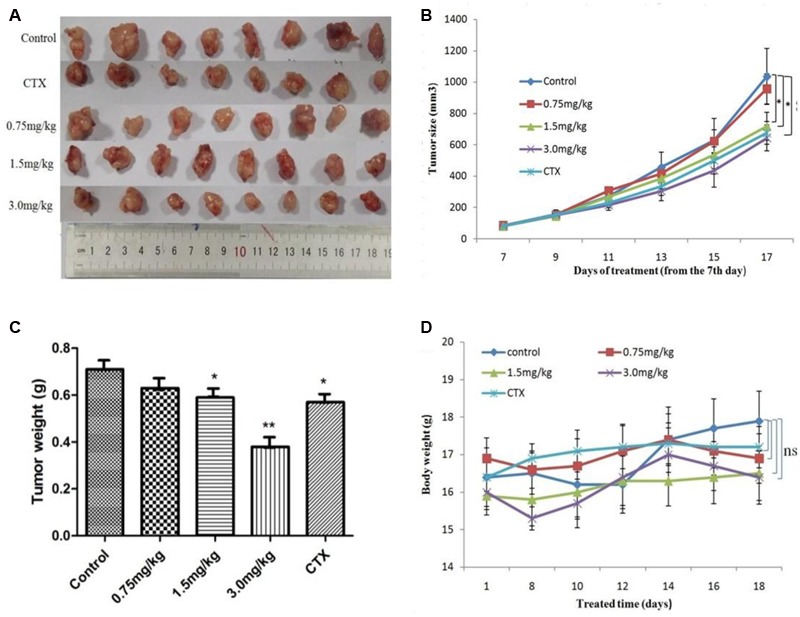
**AG36 inhibits MCF-7 xenograft growth in nude mice. (A)** Forty female BALB/c nude mice received an injection of MCF-7 cells and were divided into four groups. AG36 and CTX were administered at a dose of 0.75, 1.5, 3.0, and 25 mg/kg every other day for a total of six injections. On day 17, mice were sacrificed and tumor xenografts were excised completely from tissues. Statistical analyses demonstrated the tumor volume **(B)**, tumor weight **(C)**, and body weight **(D)** of AG36 treated and control group. ^∗^*p* < 0.05 and ^∗∗^*p* < 0.01 vs. DMSO group (*n* = 8).

After revealing the antitumor potential of AG36 in breast cancer cells *in vitro*, the antitumor effects of AG36 were also observed *in vivo*. MCF-7 cells were subcutaneously inoculated into the right anterior armpit of nude mice for 7 days, the mice were assigned to five groups randomly: control treated group with PBS, AG36 treated group (0.75, 1.5, and 3.0 mg/kg body weight, i.p. every 2 days), and CTX (cyclophosphamide) treated group (25 mg/kg body weight, i.p. every 2 days). On the last day of AG36 treatment (day 17 post-tumor injection), the tumor volumes significantly reduced compared with the control group (**Figures 7A,B**). Compared with control, AG36 at the concentrations of 1.5 and 3.0 mg/kg significantly decreased the mean tumor weight (*p* < 0.05 and *p* < 0.01) (**Figure 7C**). AG36 showed no detectable toxicity in all the groups since there were no statistically significant effects on body weight (**Figure 7D**), behavior, and appearance between the AG36 treated groups and control group. In **Table 1**, compared with control group, kidney index of the treated groups showed no significant difference, but spleen and liver index is significantly higher, which means that AG36 may improve the body immunity, while have some toxicity on liver.

**Table 1:** Effects of AG36 treatment on the liver, kidney, and spleen index of tumor-bearing mice.

Group	Dose (mg/kg)	Liver index	Kidney index	Spleen index
Control	–	42.4 ± 3.2	12.5 ± 3.2	4.6 ± 0.7
CTX	25.0	48.6 ± 2.7^∗∗^	13.7 ± 4.5	4.2 ± 0.4
AG36 (0.75 mg/kg)	0.75	48.5 ± 8.7^∗∗^	13.3 ± 4.2	6.0 ± 0.9^∗∗^
AG36 (1.5 mg/kg)	1.50	50.4 ± 3.9^∗∗∗^	13.1 ± 4.1	6.1 ± 1.1^∗∗^
AG36 (3.0 mg/kg)	3.00	52.6 ± 4.3^∗∗∗^	13.4 ± 4.4	6.7 ± 0.9^∗∗∗^

*^∗^*P* < 0.05, ^∗∗^*P* < 0.01, and ^∗∗∗^*P* < 0.001 compared with control group.*

## Discussion

Most breast cancers are diagnosed as ductal invasive carcinomas. The expression of estrogen receptor (ER), progesterone receptor (PR), and human epidermal growth factor receptor 2 (HER2) play crucial roles in ductal-derived breast cancer classification, diagnose, and treatment (Sorlie et al., 2001). In this study, we tested the antitumor activity of AG36 against three different subtype breast tumor cells namely MCF-7 (ER positive, HER2 negative), SK-BR-3 (ER negative, HER2 positive), and MDA-MB-231 (ER, PR, and HER2 negative).

Apoptosis is a genetically controlled cell-death process and plays a central role in cancer successful therapy (Bunz, 2001). It is reported that a wide variety of natural substances have been recognized to have the ability to induce apoptosis in various tumor cells of human origin (Taraphdar et al., 2001), which is regarded as a preferred way of cancer management (Hengartner, 2000; Hsu et al., 2004). In this study, AG36 could decrease the cell viability of MCF-7, MDA-MB-231, and SK-BR-3 cells in a dose- and time-dependent manner. AG36 showed more cytotoxic activity against MCF-7 cells than MDA-MB-231 cells and SK-BR-3 cells indicating that AG36 may have selective cytotoxic against ER positive breast cancer cells, which need to be further testified. At high doses, AG36 increased the proportions of G2/M cells in MCF-7, MDA-MB-231, and SK-BR-3 cells, the proportions of G1 cells were decreased accordingly. Cell cycle arresting at G2/M checkpoint can trigger apoptosis of cancer cells (Pu et al., 2002; Chao et al., 2004). The phosphorylation of Cdc2/cyclin B kinase can regulate the transition between the G2 phase and mitosis (Yarden et al., 2002), which then leads to cell arrest at the G2/M boundary without progressing to mitosis. If the DNA damage checkpoint can not be activated, the chromosomes will irreversibly rearrange and lose genomic integrity. Further investigations on the effect of AG36 on G1 and G2 cell cycle regulating proteins cyclin D1 and B1 is warranted (Fang et al., 2012). Cyclin D1 is a critical regulator essential for G1 phase progression and has a causative role in breast cancer formation (Baldin et al., 1993; Yu et al., 2001). Our study indicated that the treatment of AG36 decreased the expressions of cyclin B1, cyclin D1 in MCF-7 cells and decreased the expressions of cyclin B1 in MDA-MB-231 cells and SK-BR-3 cells, respectively. These results suggested that AG36 could block breast cancer cells proliferation via modulating cell cycle associated proteins and arresting cells in the G2/M phase.

We found the Bax/Bcl-2 ratio increased in AG36-treated MCF-7, MDA-MB-231, and SK-BR-3 cells. It is well known that the increasing of Bax/Bcl-2 ratio will cause the loss of mitochondrial membrane potential and release of cytochrome *c*, subsequently activate caspase-9, thus ultimately activate the common downstream apoptosis effector caspase-3. In our study, AG36 increased the expression of cleaved-caspase-3 and -8 but did not significantly increase the caspase-9 expression in MCF-7, MDA-MB-231 cells. In SK-BR-3 cells, AG36 increased the protein expression of cleaved caspase-3, -8 and -9, but only cleaved caspase-3 was increased significantly. Which means AG36 activated the mitochondria dependent intrinsic caspase-9 pathway together with death receptor-mediated extrinsic caspase-8 pathway and finally activated the ultimate cleaved caspase-3. Caspase-8 inhibitor z-IETD-fmk could effectively inhibit AG36-induced MCF-7 cell death. These results suggested that except for the mitochondria dependent cytochrome *c* intrinsic pathway, the death receptor-mediated extrinsic caspase-8 pathway may play a more essential role in AG36 induced apoptosis in MCF-7 cells.

The extrinsic apoptotic pathway involves a super family of death receptor ligands such as tumor necrosis factor alpha (TNF-α), TNF-related apoptosis inducing ligand (TRAIL) and FAS (Ashkenazi and Dixit, 1998). In breast cancer cells, TNF-α plays a key role in inflammation and cell apoptosis (Baud and Karin, 2001; Mathiasen et al., 2001; Jin and EI-diery, 2005). TNF-α exerts its biological functionality by binding two membrane receptors, tumor necrosis factor receptor 1 (TNFR1) and tumor necrosis factor receptor 2 (TNFR2) (Mathiasen et al., 2001). The majority of TNF signaling pathways are attributable to TNFR1, which can bind both membrane bound and soluble TNF whereas TNFR2 can only be activated by membrane bound TNF (Mathiasen et al., 2001). It is also well known that the FasL/Fas-signaling mediated death receptor apoptotic pathway is a potential target of antitumor therapy (Ziegler and Kung, 2008). In our study, it showed that the Fas, FasL, and TNFR1 levels in MCF-7 cells increased significantly with the increase of AG36 concentration. DR4 and DR5 are TRAIL receptors, their functional activity requires their physical association with lipid rafts, which serve as plasma membrane platforms for DR initiated signals in the formation of efficient DISCs (Scheel-Toellner et al., 2002; Mérino et al., 2007; Lim et al., 2011). The redistribution of DRs within the plasma membrane in lipid rafts plays an important role in TRAIL-induced apoptosis (Scheel-Toellner et al., 2004; Psahoulia et al., 2007). Yan et al. reported that TRAIL failed to induce the redistribution of DR4 or DR5 in lipid rafts and may thus explain the reason why breast cancer cells are resistant to TRAIL (Vanoosten et al., 2005). In the present study, with the increase of AG36 concentration, DR5 levels in MCF-7 cells didn’t change significantly. In MDA-MB 231 cells, AG36 increased the levels of FasL/Fas and DR5 significantly, whereas levels of TNFR1 were almost unchanged. In SK-BR-3 cells, AG36 increased Fas and FasL protein expressions but didn’t significantly affect the levels of DR5 and TNFR1. These results suggested that different breast cancer cells activated the death receptor-mediated extrinsic caspase-8 pathway through different receptors. To further dissect the antitumor mechanism of AG36, the *in vivo* experiments were carried out in xenograft animal model. After treated with AG36, the growth of MCF-7 breast xenografted tumors in the nude mice was significantly inhibited with no significant body weight loss. As the results described above, AG36 treatment (1.5 and 3.0 mg/kg/day) and positive control CTX (25 mg/kg) for 17 days exhibited apparent anti-tumor effect with some toxicity on liver when compared with control group.

## Conclusion

In summary, the findings presented in this report demonstrate that AG36 can inhibit cell survival and proliferation of MCF-7, MDA-MB-231, and SK-BR-3 cells. The anti-cancer effect was mainly mediated by cell cycle arresting, increasing ratio of Bax to Bcl-2 and cytochrome *c* releasing. The study also revealed that AG36 mediated anti-cancer effect of different breast cancer cells via different death receptors-mediated apoptosis and caspases in a carefully controlled cascade. The *in vivo* studies showed that AG36 significantly inhibited the growth of MCF-7 xenograft tumors in BALB/c nude mice comparing with control. These obtained findings may provide further insights for AG36 as a potential human breast cancer therapy.

## Ethics Statement

All animal experiments were carried out strictly in accordance with international ethical guidelines and the National Institutes of Health Guide concerning the Care and Use of Laboratory Animals. The experiments were approved by the Institutional Animal Care and Use Committee of PLA General Hospital. Female BALB/c nude mice were supplied by Beijing Vital River Laboratory Animal Co. Ltd. (Beijing, China). No endagered animal species were used in the study.

## Author Contributions

L-HM was involved in the project design, carried out most of the experiments, and drafted the manuscript. Y-NW, X-ZD, YH, JZ, and LL participated in the molecular, biochemical, and cell biological work. D-XW contributed to the animal experiment and data analysis. PL conceived and designed the experiments. All authors read and approved the manuscript finally.

## Conflict of Interest Statement

The authors declare that the research was conducted in the absence of any commercial or financial relationships that could be construed as a potential conflict of interest.
